# Psychotherapy with a 3-Year-Old Child: The Role of Play in the Unfolding Process

**DOI:** 10.3389/fpsyg.2016.02021

**Published:** 2017-01-04

**Authors:** Silvia Salcuni, Daniela Di Riso, Diana Mabilia, Adriana Lis

**Affiliations:** Dipartimento di Psicologia dello Sviluppo e della Socializzazione, Università degli Studi di PadovaPadova, Italy

**Keywords:** psychodynamic psychotherapy, single case study, process-outcome research, child, play, verbal productivity

## Abstract

Few studies have investigated the outcomes and process of psychodynamic psychotherapies with children. Among the limited number of studies, some only paid attention to play and verbal production, as they are fundamental aspects in assessing the psychotherapy process. This paper focuses on an empirical investigation of a 3-year, once-a-week psychodynamic psychotherapy carried out with a 3-year-old girl. A process-outcome design was implemented to evaluate play and verbal discourse in in the initial, middle, and final parts of 30 psychotherapy sessions. Repeated measurements of standardized play categories (the Play Category System and the Affect in Play Scale—Preschool version) and verbal discourse (Verbal Production) were analyzed. To increase the clinical validity of the study, data from the assessment phase and vignettes from the sessions were reported to deepen the patient’s picture during the unfolding therapy process. Parent reports before and after the therapy were also included. Empirically measured changes in play and verbal production were fundamental in evaluating the young patient’s psychotherapy process. Verbal production and discourse ability progressively increased and took the place of play, which instead became more symbolic. Developmental issues as well as psychotherapy’s influence on the patient’s change, were discussed in relation to the role of play in enhancing the development of verbal dialog and the expression of the child’s emotions, needs, and desires.

## Introduction

Recently, increasing interest has been devoted to the assessment of “operationally defined” markers of the psychodynamic psychotherapy process to alleviate children’s distress (Delgado, 2008), underlining the importance of introducing well-validated and standardized research instruments to study clinical processes (Midgley and Kennedy, 2011; Yanof, 2013). The empirical support for psychodynamic psychotherapy with children has been limited as compared with adults (Abbass et al., 2013); few studies have focused on the outcomes and process of psychodynamic psychotherapy with children, and just a small number of them have paid attention to methodological issues (Weisz and Hawley, 2002; Kennedy, 2004), including mixed empirical evidence (Abbass et al., 2013).

In psychodynamic psychotherapy for children, the emphasis is not only placed on verbal communication but also on non-verbal communication, by considering the child’s developmental level to facilitate therapeutic relationships throughout the play, drawing and dialog (Shirk and Karver, 2003; Kernberg et al., 2012). The development of play is an important milestone in childhood. Play holds a crucial role in providing a safe, caring, protective, confidential, and containing space where children can recreate themselves and their painful experiences through a process of self-cure (Winnicott, 1942; Erikson, 1963; Landreth, 2002; Bratton et al., 2005; Campbell and Knoetze, 2010). Pretend play is the best way of expressing thoughts and emotions (Kernberg et al., 1998; Halfon et al., 2016) as well as mitigating fears and anxieties (Harris, 2000; Russ, 2004; Yanof, 2013). It is characterized by the use of fantasy, a level of organization and a standard of comfort (Russ, 2004; Yanof, 2013). Fantasy is the process of make-believe, an essential behavior the child engages in during pretend play; organization helps the child to structure pretend play into a story and to utilize cause-and-effect thinking; and comfort is used to assess the ease and pleasure in the engagement in play.

All of these milestones make it easy to understand why play has been considered a preferential way of exploring the inner world of child psychoanalytic therapy since the 1930s, when Melanie Klein and Anna Freud used play techniques to help their young (not fluently speaking) clients to express thoughts, emotions, and feelings. Starting from these beginnings, play has been considered as (a) the primary expressive medium in child psychotherapy to hold meaningful therapeutic value (Bratton et al., 2005; Barish, 2009; Campbell and Knoetze, 2010), (b) a natural co-constructed means of communication between the child and the therapist, and (c) an useful therapeutic technique to help the child work through different meanings and managing stressful emotions (Russ, 2004; Yanof, 2013).

Similar to pretend play, drawing and verbal communication are natural childhood manners of expression, which provides a space where children can feel comfortable (Brems, 2008; Midgley et al., 2009; Pace et al., 2015; Capella et al., 2016). Verbal production, finally, has an important role in assessing the psychological/mentalistic lexicon formed by terms referring to mental states. Its appearance is considered an important indicator of early understanding of mind as well as one’s and others’ internal worlds, and a precursor of subsequent meta-representational capacity (Bartsch and Wellman, 1995; Baumgartner et al., 2000; Ornaghi et al., 2010). Longitudinal studies suggest that in non-clinical children from the age of 2, the child should be able to use a mentalistic lexicon when referring to perception, complex feelings, and social emotions. At around 3 years, a cognitive psychological lexicon appears concerning internal states related to beliefs, wishes and imagination (Ornaghi et al., 2010).

The principal aim of this paper is to investigate therapeutic change in play, using operationalized and validated measure systems, and to explore its relationship with drawing and dialog in a psycho-dynamically oriented psychotherapy with a 3-year-old girl. Play, drawing and verbal production were fundamental aspects in assessing the therapeutic change. Improvements in psychological complexity and representational skills in terms of symbolic play were expected, given their importance to children of the patient’s age and to the sophistication of verbal skills (Fein, 1987).

The present work was an observational study, corresponding to a level-5 study following the hierarchy of evidence provided by Midgley and Kennedy (2011); it provides a detailed discussion of a clinical single case using a process-outcome design. Its aim is to analyze the change during the psychotherapy, using outcome measures to provide a general view on the patient’s functioning through comparison between the assessment phase and the outcome evaluation. In order to provide more robust testing, different types of instruments were used in the assessment and outcome phases, with each one revealing different aspects of specific constructs (Cheng, 2001).

Improvements in play measured during spontaneous play moments and in drawing and dialog within the therapeutic sessions were hypothesized to support more accurate competencies in managing, naming and modulating emotions as well as in talking about “self-inner states.” Moreover, since the positive effect of children’s involvement in non-verbal activities on the verbal expression of inner dialog, measured by verbal production, was expected to progressively increase during therapy and progressively substitute the massive use of play and other activities (e.g., van Nijnatten and van Doorn, 2013). A multimethod approach was used to gain incremental clinical validity in understanding the case.

The following paragraph first includes the patient’s – Sarah – referral by her parents and the therapy aims. Then, the instruments and results of play are presented to compare three phases of the treatment. The analysis shifts to measures of play and verbal expression and to therapeutic change during the different stages of the psychotherapy (T1, T2, and T3). Finally, conclusions are drawn to take stock of Sarah’s case to integrate the outcomes with the change during the therapy.

## Materials and Methods

Psychotherapy was held in the clinical centre of the University of Padova. Following the service of good practices and the Italian law about privacy and data confidentiality (n°196/03), written and informed parental consent was asked and obtained for video and audio sessions recording, as well as for the participation in the research.

The treatment lasted 3 years and consisted of 55 once-a-week sessions, which were audio-recorded and fully transcribed with the parents’ informed consent. In the present work, 30 sessions were scored and analyzed: 10 from the first phase (T1), 10 from the central phase (T2), and 10 from the last phase (T3) of therapy. Therapy was held by a female therapist in training who received weekly supervision from highly competent clinicians at the University clinical centre. During the assessment and outcome phases, the Affect in Play Scale—Preschool version (APS-P; Russ, 2004) was administered to Sarah at the beginning of, about halfway through and at the very end of the therapy, respectively, to assess her cognitive and emotional expression and to observe her level of pretend play.

## The Case of Sarah

Sarah was a 3-year-old Italian child who was referred by her parents. Sarah came from an intact family with middle socioeconomic status. Her mother had graduated and worked as an employer; her father was a teacher at an elementary school. They came from intact families and did not report any specific traumatic events in their life.

One year before the present referral, Sarah, at the age of two, was referred by her parents for speech difficulties, oppositional behavior and sleep problems. During that occasion, only parenting support and advice were offered to help the parents better understand and manage Sarah’s difficulties.

One year later, Sarah’s parents re-contacted the centre asking for help because Sarah’s symptoms were back and because they complained about deterioration in some areas. First, the therapist met Sarah’s parents again, without the child, to assess how they perceived her daughter and their functioning toward the child, and the therapist adjourned the child’s anamnestic history. Sarah’s parents were particularly worried about their little daughter. Regarding Sarah’s language impairment, the parents reported Sarah’s decrease in verbal ability (stuttering, changing letters in words and difficulties with naming objects) along with a general regression to baby talk. Sarah’s oppositional behavior had also relapsed, as Sarah often seemed upset and had tantrums. In these situations, Sarah’s parents felt that they were unable to calm down and relax Sarah, and they felt distressed, powerless, and inadequate. Moreover, they reported a regression in several of Sarah’s competencies, concerning feeding (she wanted her mother to feed her), social inhibition (Sarah looked more isolated and less interested in her peers than before) and aspects of separation anxiety (she needed her parents to play with and stay next to her most of the time). A psychodynamic assessment was done using Anna Freud’s developmental lines. In particular, difficulties and regressions were found in many developmental lines at the beginning of the therapy. Following “from dependency to emotional self-reliance and adult object relationships,” Sarah showed a regression to a more dependent phase of life and was unable to stay alone even for few minutes to play or draw, and always asked for her mother’s presence (regression in the line from the Body to the Toy and from Play to Work). At the preschool, she asked for the teacher’s company and showed more difficulties in behaving and playing with other children (regression in the line from Egocentricity to Companionship). She had previously developed the ability to eat using a spoon and a fork, but at the moment of the assessment, she seemed unable to eat alone and was always asking for maternal care and help; in this case, a strong regression in the developmental line “From Suckling to Rational Eating” was found. At the same time, at the age of three, she had begun to wash her face, prefer and choose her clothes and try to dress alone, but at the moment of assessment, her regressive behavior showed she was unable to do anything in autonomy (From Irresponsibility to Responsibility in Body Management).

The therapist observed that the parents were only able to report negative descriptions when talking about their little girl. There was no pleasure or positive affection to share about their daughter. To the therapist, they appeared quite rigid, anxious about the adequacy of Sarah’s behaviors and requests, and to not always be able to understand or support Sarah’s developmental needs or understand their child in connection with her real age and developmental stage. They tended to consider their daughter as a “little adult” whose behaviors were too “childish.” Typically, their interactions with Sarah were about normative conduct: “You are a grown-up girl. Help yourself. Behave yourself. Keep sitting in a good manner.” The interactions surrounding the play were like, “You are playing too much; now, try to draw something nice for your mom.”

During the assessment phase, Sarah showed the impairment her parents had declared, highlighting a state of emotional distress and a sense of emptiness and loneliness. She looked sad, showed poor facial expressions, showed no interest in exploring the room or in playing with toys and did not talk to the therapist. However, she was able to stay alone and was eager to stay with the therapist and to follow her suggestions for interaction. According to the therapist, Sarah showed a disposition (according to her young age) to “use” the therapeutic space and the therapeutic relationship for her developmental issues, and to use the therapist as a “real relational object” to identify and interact with. She absolutely needed her own space (the therapy) to find a new model of relationship in which to express her developmental needs and emotions, without rigid requests of adjustment to be a well-behaved “grown-up girl.”

The therapist thought about what would be the best way to help this family, especially Sarah, and decided to offer a parallel path: to continue working with parents and, at the same time, to offer personal individual treatment to Sarah. The latter was motivated by Sarah’s psychodynamic assessment, which highlighted both Sarah’s indication for psychological support (symptoms and regressions) and quite a stable sense of self to gain better adjustment throughout individual psychoanalytic “developmental help,” as suggested in psychoanalytic training schools for children aged 3 to 5 years old (pre-latency cases). This double therapeutic intervention was accepted by the parents, and they started to have regular meetings twice a month and to support a weekly individual treatment with their child.

The aim of working with Sarah’s parents was to help Sarah’s parents to support their parenting function during Sarah’s therapy. The therapist worked hard to create and improve on her strong working alliance with Sarah’s parents, never making them feel inadequate while at the same time increasing their parenthood abilities to give meaning to Sarah’s behaviors and to keep her needs in mind (also telling them specific vignettes about what Sarah was doing in therapy and connecting the vignettes with Sarah’s behavior they reported at home), to help Sarah reach better adjustment and wellbeing. The therapeutic goals for Sarah focused on behavioral regulation, decreasing inhibition and separation anxiety symptoms as well as modulating her oppositional behavior and increasing her emotional expression. The therapy was also aimed at helping Sarah to acquire relational skills and interest in others to allow her to face new situations more adequately. As in every psychodynamically oriented psychotherapy, the therapeutic relationship played a basic role in the therapy process; play and dialog were used to support the quality of the therapeutic relationships and motivation as well as to reach the therapy goals.

This paper focused on the specificity of the child’s treatment.

### Procedure

The present work includes: (a) a comparison of psychological assessments and therapy outcomes through the Affect in Play Scale—Preschool version, which was administered in line with its standardized procedure (APS-P; Russ, 2004); (b) a descriptive analysis about how periods devoted to drawing, playing and dialog changed during the unfolding of the therapy; and (c) a therapeutic change analysis along three therapy sessions, revealed by measures of verbal expression and play and applied on spontaneous play during the sessions. Play was assessed with the adaptations of APS-P and the Bornstein Play Category System at the initial, central, and final phases of selected therapy sessions. Frequencies and type of verbs referring to the state of being, behavior and state of mind were applied as measures of psychological/mentalistic lexicon during each of the 30 sessions, as suggested by Camaioni et al. (1998).

### Tools Description

Affect in Play Scale-Preschool version (APS-P; Russ, 2004). The APS-P (Russ, 2004; Kaugars and Russ, 2009) is a semi-structured, empirically validated, individually administered 5-min play task that assesses affective and cognitive dimensions of play (Russ, 2004). Standardized instructions and scoring were provided. The child was invited to play with a set of plastic and stuffed toys, including animals (bear, shark) and objects (car, small cups, a “hairy” rubber ball) intended to elicit a range of emotional expressions such as aggression (e.g., a shark). With regard to cognitive scores, organization assesses the quality, complexity, and coherence of the play narrative, with scores ranging from (1) unrelated events, no cause and effect, to (5) integrated plot with a beginning, a middle part and a conclusion. Elaboration refers to the variety and complexity of elements used in the story themes, such as facial expressions, sound effects and characters’ development, from (1) very few details and simple themes with no embellishment, to (5) much embellishment across many dimensions such as details, sound and voice effects and facial expressions. Imagination assesses fantasy and the number of transformations (e.g., using one thing as another) in the play, ranging from (1) no symbolism, no fantasy, to (5) many transformations and fantasy themes. Comfort measures the child’s ability to get involved in the play task and his or her enjoyment of the play, ranging from (1) reticent, distressed, to (5) very involved and enjoying the play. The expression of affects was coded as regarding the Frequency of Affect Expression, which was used during the play session. Affect is scored when an affect theme is expressed in the play. Affect scores can be positive (e.g., nurturance/affection) or negative (e.g., aggression), and they can be summed to form the total affect.

An adaptation of the APS-P using the toys available in the therapy room was used to assess therapeutic change during the therapy by measuring cognitive and affective variables in the spontaneous play in terms of presence and quality (positive or negative) of affect expression as well as the cognitive level of play organization, elaboration, imagination, and comfort. The scores on the APS-P in its regular and adapted use in clinical sessions were calculated by two independent judges – the therapist and a Ph.D. student who were both trained in the APS-P and were blind about which phase the sessions were from. The agreement between the two judges was satisfactory.

An adaptation of the Bornstein Play Category System (PCS; Bornstein and O’Reilly, 1993; Bornstein, 2007) was used to assess Sarah’s spontaneous play with toys like a dollhouse, a camping tent, and cups, all of which were available in the therapy room. According to this system, play levels are empirically devised to detect the progressive nature of play across the first years of life. Levels 1–4 includes categories of exploratory play, while Levels 5–8 includes categories of symbolic play. A brief description of the levels is reported in **Table 1**. The play was coded from videotapes in accordance with the mutually exclusive and exhaustive eight play category levels and a default (no-play) category for each level, and the absolute frequency was calculated. The PCS looked likely to represent a useful instrument in assessing Sarah’s play because Sarah’s level of symbolic play seemed to be scarce at the beginning of therapy, compared to children of her age, and the PCS can give a more detailed evaluation regarding levels of play, from exploratory to symbolic levels. The play categories were assessed in Sarah’s spontaneous play during therapeutic sessions, considering separately the initial, central, and final phases of therapy.

**Table 1:** Trends of play and verbal sophistication from T1 to T3.

	T1		T2		T3		*F*	*p*	*Post hoc*
						
Borstein Play Category System	*M*	*DS*	*M*	*DS*	*M*	*DS*			
*Unique functional activities*	3.00	4.19	0	0	0	0	–	–	–
*Inappropriate combined activities*	0	0	0	0	0	0	–	–	–
*Appropriate combined activities*	2.30	3.97	2.20	4.57	1.40	4.43	0.48	0.62	–
*Transitional play*	2.50	5.32	0	0	0	0	–	–	–
*Symbolization of the self*	3.10	3.67	0	0	0	0	–	–	–
*Symbolization of others*	6.90	10.26	0	0	0.50	0.71	–	–	–
*Symbolization sequence*	6.00	6.59	8.80	12.20	9.90	8.02	3.60	<0.05	T3 = T2 > T1
*Symbolization replacement*	1.80	2.53	4.20	5.65	5.90	5.35	3.70	<0.01	T3 > T2 > T1
Adaptation of Russ APS-P									
*Organization*	1.00	0.98	1.00	1.05	3.00	1.41	4.20	<0.05	T3 > T1 = T2
*Elaboration*	1.00	0.76	1.00	0.79	2.00	1.02	2.43	0.13	–
*Imagination*	1.00	0.89	2.00	0.78	3.00	0.95	4.00	<0.05	T3 > T2 > T1
*Comfort*	1.00	1.03	2.00	0.09	3.00	0.87	4.01	<0.05	T3 > T2 > T1
*Positive affective expression*	70.80	23.04	78.70	36.71	65.47	31.46	2.61	0.09	–
*Negative affective expression*	33.70	21.75	72.40	40.35	47.27	32.38	6.13	<0.01	T2 > T1 = T3
*Total affective expression*	121.70	42.04	199.30	86.93	121.80	51.61	5.016	<0.05	T2 > T1 = T3
Camaioni – Verb development									
*Behavior*	107.8	48.28	140.3	65.02	180.3	54.32	3.65	<0.05	T3 > T2 = T1
*State of Mind*	38.6	20.14	84.2	47.39	105.7	48.23	6.13	<0.01	T3 > T2 > T1
*State of Being*	34.5	19.15	56.3	36.10	76.9	49.65	2.62	0.09	–


#### Verbal Production

Every language includes very different types of words; of specific interest are words conveying emotions, feelings, wishes, thoughts, and beliefs, all of which are included in what is defined as the psychological lexicon, which is formed by terms referring to mental states. Its appearance in children around 3 years old is considered an important indicator of early understanding of the mind as well as one’s and others’ internal worlds as well as a precursor of the subsequent meta-representational capacity (Bartsch and Wellman, 1995; Baumgartner et al., 2000; Ornaghi et al., 2010). To empirically identify this developmental progression toward a psychological/mentalistic lexicon, following Camaioni et al. (1998), verbal production was classified into three categories referring to the acquisition of an increased psychological complexity: (a) state verbs, which are verbal forms that do not refer to mental states such as “there is, there are”; (b) behavior verbs, which are verbal forms that express concrete actions such as eat, walk and read; and (c) mental verbs, which are verbal forms that include all verbal expressions that are more connected with the cognitive and emotional components of thoughts in both positive and negative terms – they not only include feelings and thoughts but also volition states, moral judgments and acknowledgments of abilities. Two blind judges independently scored the test, and the inter-rater reliability was satisfactory.

### Data Analysis

The percentages of time devoted to playing, dialog or drawing/other activities were monitored in each session, namely at T1, T2, and T3, to evidence the differing quality of activities that unfolded during the therapeutic session over time. Descriptive statistics and MANOVA for repeated measures were used to analyze the results, with respect with therapeutic change during the three periods, focusing on the quality of spontaneous play in terms of cognitive, affective, and concrete/symbolic modalities of expression. Visual graphics were reported for significant variable changes, specifically assessment and outcome changes as well as the changes within T1, T2, and T3.

## Results

### Comparison between Assessment and Outcome Scores

**FIGURE 1:**
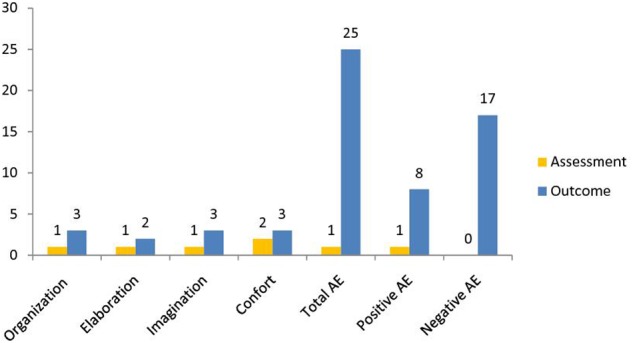
**Mean, APS-P cognitive and affect scores at assessment and outcome**.

According to the APS-P, cognitive expression improved from the assessment to the outcome (**Table 1**; **Figure 1**). Cognitive expression in play was also assessed by comparing Sarah’s results with normative scores of the Italian sample (children between 4 and 5 years; Mazzeschi et al., 2016). The Assessment scores were between the 30th and 40th percentiles. The outcome scores increased, reflecting relevant improvements in cognitive functioning. Elaboration and comfort increased through the third quartile (60th and 70th percentiles, respectively), while the organization and imagination scores increased even through the fourth quartile (90th and 95th, respectively), thus reflecting higher scores compared to those of normative sample.

Sarah’s APS-P affect scores were also assessed (**Table 1**; **Figure 1**), by comparing Sarah’s results with normative scores of the Italian sample (children between 4 and 5 years; Mazzeschi et al., 2016). Sarah’s assessment scores were very low – within the first quartile (between the 10th and 20th percentiles). However, Sarah’s scores increased after therapy, reflecting relevant improvements in her emotional understanding and expression.

Below are examples of Sarah’s verbalizations during the APS-P in T1 and T3:

T1: The animals are doing things. They eat.
*T3*: The shark would like to bite the animals. They are very worried. They need help from Daddy.

Total affect increased, going up through the fourth quartile (80th). More specifically, Sarah’s positive affect score was around the median (60th percentile), while her negative affect score was within the fourth quartile (95th percentile), thus reflecting higher scores compared to those of normative sample.

### Change in the Measures of Play and Verbal Discourse in the Different Psychotherapy Periods

**FIGURE 2:**
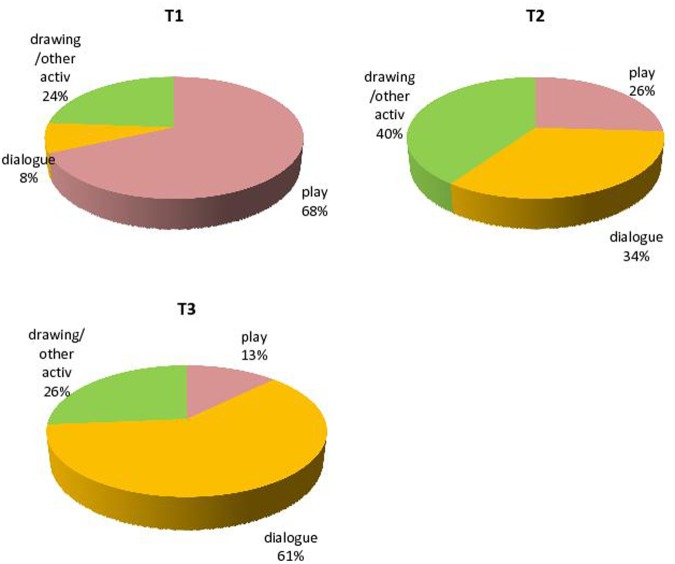
**Play, dialogue and drawing/other activities in T1, T2, and T3**.

In order to analyze Sarah’s activities during her therapy sessions, three categories were separately counted in terms of “time” dedicated to: (a) play, in terms of Sarah’s verbal and non-verbal expression during play with toys, using an adaptation of APS-P; (b) dialog, or Sarah’s speech during activities that were different from play; and (c) drawing/other activities, such as book reading and storytelling. Play, dialog and drawing/other activities were measured as percentages regarding the three considered therapeutic periods (T1, T2, and T3). More specifically, drawing/other activities and dialog progressively became more frequent during sessions with Sarah, whereas play decreased (**Table 1**; **Figure 2**).

**FIGURE 3:**
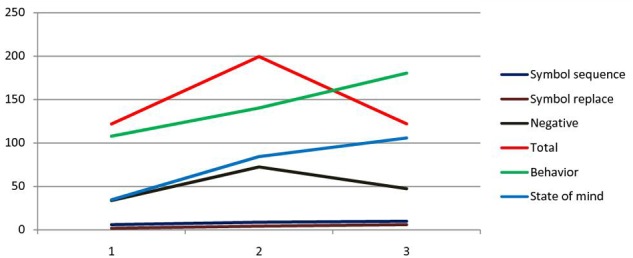
**Trends of play categories (Bornstein and O’Reilly, 1993), APS-P (Russ, 2004) and verbal expression (Camaioni et al., 1998) in the three times of psychotherapy**.

Mean play sophistication, as assessed by the adaptation of the Bornstein Play Category System from Sarah’s play with toys, improved from T1 to T3 (**Table 1**; **Figure 3**). Immature components of play, like functional activities, were replaced by more mature categories, like symbolization sequence and replacement, which started to increase significantly from the beginning to the third period of therapy. For example:

*T1: “Here, you are an elephant…he is gray…big…he eats something. I do not know…here, there is nothing to eat.*”
*T3: Mom, Dog and his son go for a walk. They go to the swimming pool (she indicates a blue piece of wood)…They have a lot of fun; the little dog was happy.*”

The means and standard deviations of the cognitive variable scores, assessed by the adaptation of APS-P Sarah’s play with toys, are shown in **Table 1**. Sarah’s cognitive scores, organization, imagination and feeling of comfort increased significantly from T1 to T3 of the therapy. With respect to affect expression assessed with APS-P during play, the means and standard deviation are presented in **Table 1**. At a qualitative level, aggression, happiness, oral and frustration were the most common affective categories in Sarah’s dialog. Total and negative affective expression increased from T1 to T2 and then decreased until the end of treatment (**Figure 3**). In the middle of therapy, due to therapeutic holding, Sarah felt more and more comfortable in expressing negative emotional states, feeling sure about finding acceptance and elaboration. This allowed Sarah to express her negative affect, probably associated with her parents’ failure to provide an adequate holding environment.

See a verbalization in T2:

T2: “This giraffe is very angry…because her friend asks her to run very fast…but she is just a little giraffe…!

The verbs most frequently used in Sarah’s dialog were those referring to behavior (Camaioni et al., 1998), followed by verbs regarding state of mind and state of being (**Table 1**; **Figure 3**). Both behavior and state of mind verbs increased significantly from T1 to T3, reflecting Sarah’s improving capacity to behave concretely and to report her inner mental states.

## Conclusion

At the end of the therapy, another developmental evaluation was done using Anna Freud’s developmental lines. Sarah reached normal development in all of the developmental dimensions that were compromised at the beginning of the therapy. The present work analyzed a good treatment outcome in a 3-year-old child, in terms of symbolic play changes. At the beginning of therapy, Sarah was not comfortable with play; thus, during T1, the therapist aimed to make Sarah feel more comfortable with playing in the therapy room. With children who are not able to play, the therapist’s goal is to help them use play as a means of self-expression and as a way of create meaning in the presence of another (Yanof, 2013). T2 represented an important phase in Sarah’s treatment. This is probably due to her acquisition of higher comfort in therapy. Due to the therapy, Sarah’s play progressively improved from exploratory to symbolic play. Moreover, Sarah’s affect expression increased, particularly negative emotions, which did not disappear but strongly decreased in the middle and then re-increased at the very end of the therapy. More important than the quantitative characteristics are the qualitative characteristics of this trend, which seem important from a clinical point of view. Sarah learned to explore and affirm the expression of her negative affect in the therapeutic setting, increasing the “bad feelings” in the middle of the clinical work. Then, she learned to manage and cope with such emotional expressions: the quantity decreased, but more importantly, the quality of the negative affect became more “workable,” and Sarah was more prone to explore and elaborate upon these feelings in her play. Progressively, Sarah expressed aggressive affects through fantasy and cognitive elaboration, which allowed an adequate expression of aggressive emotions in much more of a holding setting like the therapeutic one that, in contrast with her parents, recognized her developmental gains. This was confirmed by Sarah’s assessment/outcome results on the APS-P scores: the percentiles showed that Sarah’s results were not in the normative range for her age at the assessment phase, but at the end of the treatment, the percentiles showed that Sarah had reached the range of normal development in symbolic play. This positive psychotherapy outcome was also confirmed in the analysis of Anna Freud’s developmental line at the end of the therapy: Sarah was less disharmonic that was in line with the developmental stage she was in. The results shed light on further investigation about the process of change.

Play had a core role in this psychotherapy, by showing a link with affective expressions and verbal production. These findings are in line with Russ’s (1993, 2004) theoretical model that proposes play as being strictly connected to both cognitive and affective domains. Through play (and dialog), Sarah learned to express and modulate her feelings when referring to a wider range of emotional patterns. Specifically, her play decreased in frequency, but its quality improved concerning symbolic thoughts, cognitive and affective contents and verbal expression. Lower frequency of play allowed Sarah’s dialog to improve with regards to frequency and quality, such as supporting representation of mental states. Several scholars (Berk et al., 2006) have suggested that make-believe games are forerunners of the important capacity for forms of self-regulation, including reduced aggression, delayed gratification, civility and empathy. The improvement in verbs referring to states of mind reflects this acquisition. Higher levels of negative affect expression, compared to those positive affect, probably reflected Sarah’s feelings of not being initially recognized by her parents, who initially only reported her episodes of tantrum and oppositional behaviors without revealing her positive developmental aspects. From a more qualitative viewpoint, psychotherapy revealed its utility in terms of the decrease in symptoms and the progressive development of cognitive and affective components in Sarah’s functioning. The relationship between Sarah and her parents was very difficult in the first place. Her father and mother were not able to find pleasure in staying with their daughter, and they only reported negative descriptions and faults when talking about their daughter. However, at the end of therapy, Sarah’s mother and father acknowledged Sarah’s improvements, reporting that she showed fewer symptoms when she was at home, such as oppositional behavior.

The present study had some limits, therefore leaving some open questions. Since this is a single case study, the results cannot be generalized. The complexity examined is difficult to represent simply and briefly. The intervening outcomes may have appeared to be stronger if the researcher was more experienced. Finally, it cannot answer a large number of relevant and appropriate research questions (Hodkinson and Hodkinson, 2001), such as – specifically at developmental age – how the change is understandable in terms of psychotherapy’s contribution or the effects of natural developmental issues. Moreover, changes in the drawing activities were not directly measured or evaluated in their changes, but just for their expression in the APS-P and verb categories. More important information could be added in future research with respect with changes in typical drawing dimensions, during the unfolding of the therapy. However, this particular single case with a very little girl could be considered original and ecological because it is grounded in “lived reality” of the therapeutic exchange with a little patient, where communication passed through non-verbal more than verbal communication. Moreover, the particular combination of Sarah’s “developmental help” therapy and the “working with parents” intervention highlighted the importance of creating a working alliance web around the young patient’s suffering. This kind of work increased the effectiveness of the intervention. In this sense, the reduction in the child’s symptoms appeared to be the consequence of the double support to both the parents’ role and the child’s development.

Focusing on play and verbal development, from more concrete to more symbolic, helps us picture the inner world of a patient with a – quite typical and even difficult – immature level of functioning, and understand complex inter-relationships among diagnosis, measures and their clinical application (Salcuni et al., 2015). As Hodkinson and Hodkinson (2001) suggested, a single case study can provide “provisional truths, in a Popperian sense,” until contradictory findings or better theories are developed. Moreover, following a strong empirical approach to change through play and dialog change, this case can be considered useful to highlight the importance of an empirical approach to psychodynamic psychotherapy research with children.

## Ethics Statement

We followed the procedure suggested in our Department, in line with the university local ethical committee, asking to both Sarah’s parents for their written informed consent, Sarah is a fictional name, and all information about the child and her parents that could make this family recognizable was modified. The Clinical Service in which the study was conducted, is a recognized research centre of our university (Interdepartmental Laboratories for Research and Applied Psychology, LIRIPAC); all the studies conducted on patients followed the LIRIPAC and Department ethical guideline and procedures, based on the Italian law about privacy and confidentiality (n° 196/03); research practice and ethical procedure were discussed with the Director of the Centre and approved before the research began.

## Authors Contributions

SS followed the whole process of the manuscript, supervising it, and writing interpretative conclusions and discussion of the case report; AL supervised method and procedure: moreover, she supervised some years ago the therapist that took care of Sarah; DM wrote the introduction and, together with DDR, performed scoring of the clinical material and the data analysis and the table editing.

## Conflict of Interest Statement

The authors declare that the research was conducted in the absence of any commercial or financial relationships that could be construed as a potential conflict of interest.

## References

[B1] Abbass A. Rabung S. Leichsenring F. Refseth J. Midgley N. (2013). Psychodynamic psychotherapy for children and adolescents: a meta-analysis of short-term psychodynamic models. *J. Am. Acad. Child Adolesc. Psychiatry* 52 863–875. 10.1016/j.jaac.2013.05.014 23880496

[B2] Barish K. (2009). *Emotions in Child Psychotherapy: An Integrative Framework*. Oxford: Oxford University Press.

[B3] Bartsch K. Wellman H. M. (1995). *Children Talk About the Mind.* Oxford: Oxford University Press.

[B4] Baumgartner E. Devescovi A. D’Amico A. (2000). *Lessico Psicologico Nell’infanzia.* Roma: Carocci.

[B5] Berk L. E. Mann T. D. Ogan A. T. (2006). “Make-believe play: wellspring for the development of self-regulation,” in *Play = Learning: How Play Motivates and Enhances Children’s Cognitive and Social-Emotional Growth*, eds Singer D. G. Golinkoff R. M. Hirsh-Pasek K. (Oxford: Oxford University Press), 74–100.

[B6] Bornstein M. H. (2007). “On the significance of social relationships in the development of children’s earliest symbolic play: an ecological perspective,” in *Play and Development: Evolutionary, Sociocultural, And Functional Perspectives*, eds Gönçü A. Gaskins S. (Mahwah, NJ: Erlbaum), 101–129.

[B7] Bornstein M. H. O’Reilly A. W. E. (1993). *The Role of Play in the Development of Thought.* San Francisco, CA: Jossey-Bass.

[B8] Bratton S. C. Ray D. Rhine T. Jones L. (2005). The efficacy of play therapy with children: a meta-analytic review of treatment outcomes. *Prof. Psychol. Res. Pr.* 36 376 10.1037/0735-7028.36.4.376

[B9] Brems C. (2008). *A Comprehensive Guide to Child Psychotherapy and Counseling.* Long Grove, IL: Waveland Press.

[B10] Camaioni L. Longobardi E. Bellagamba F. (1998). Evoluzione dei termini di stati mentali nelle storie di fantasia scritte da bambini in età scolare. *Etàevolutiva* 60 20–29.

[B11] Campbell M. M. Knoetze J. J. (2010). Repetitive symbolic play as a therapeutic process in child-centered play therapy. *Int. J. Play Ther.* 19 222 10.1037/a0021030

[B12] Capella C. Lama X. Rodríguez L. Águila D. Beiza G. Dussert D. (2016). Winning a race: narratives of healing and psychotherapy in children and adolescents who have been sexually abused. *J. Child Sex. Abuse* 25 73–92. 10.1080/10538712.2015.1088915 26789104

[B13] Cheng E. W. (2001). SEM being more effective than multiple regression in parsimonious model testing for management development research. *J. Manag. Dev.* 20 650–667. 10.1108/02621710110400564

[B14] Delgado S. V. (2008). Psychodynamic psychotherapy for children and adolescents: an old friend revisited. *Psychiatry* 5 67–72. 19727254 PMC2686640

[B15] Erikson E. (1963). *Children and Society.* New York, NY: Norton.

[B16] Fein G. G. (1987). “Pretend play: creativity and consciousness,” in *Curiosity, Imagination, and Play: On the Development of Spontaneous Cognitive Motivational Processes*, eds Gørlitz D. Wohlwill J. F. (Hillsdale, NJ: Erlbaum, Inc), 81–304.

[B17] Halfon S. Çavdar A. Orsucci F. Schiepek G. K. Andreassi S. Giuliani A. (2016). The nonlinear trajectory of change in play profiles of three children in psychodynamic play therapy. *Front. Psychol.* 7:1494 10.3389/fpsyg.2016.0149 PMC5056176 27777561

[B18] Harris P. L. (2000). *The Work of the Imagination.* Hoboken, NJ: Blackwell Publishing.

[B19] Hodkinson P. Hodkinson H. (2001). “The strengths and limitations of case study research,” in *Proceedings of the Learning and Skills Development Agency Conference at Cambridge* Vol. 1 Cambridge, 5–7.

[B20] Kaugars A. S. Russ S. W. (2009). Assessing preschool children’s pretend play: preliminary validation of the affect in play scale-preschool version. *Early Educ. Dev.* 20 733–755. 10.1080/10409280802545388

[B21] Kennedy E. (2004). *Child and Adolescent Psychotherapy: A Systematic Review of Psychoanalytic Approaches.* London: North Central London Strategic Health Authority.

[B22] Kernberg P. F. Chazan S. E. Normandin L. (1998). The children’s play therapy instrument (CPTI): description, development, and reliability studies. *J. Psychother. Pract. Res.* 7 196–207. 9631341 PMC3330503

[B23] Kernberg P. F. Ritvo R. Keable H. (2012). Practice parameter for psychodynamic psychotherapy with children. *J. Am. Acad. Child Adolesc. Psychiatry* 51 541–557. 10.1016/j.jaac.2012.02.015 22525961

[B24] Landreth G. L. (2002). Therapeutic limit setting in the play therapy relationship. *Prof. Psychol. Res. Pr.* 33 529 10.1037/0735-7028.33.6.529

[B25] Mazzeschi C. Salcuni S. Di Riso D. Chessa D. Delvecchio E. Lis A. (2016). *E tu Giochi? La Valutazione del Gioco Simbolico in età Evolutiva: l’Affect in Play Scale.* Milano: Franco Angeli.

[B26] Midgley N. Anderson J. Grainger E. Nesic-Vuckovic T. Urwin C. (2009). *Child Psychotherapy and Research: New Approaches, Emerging Findings.* Abingdon: Routledge.

[B27] Midgley N. Kennedy E. (2011). Psychodynamic psychotherapy for children and adolescents: a critical review of the evidence base. *J. Child Psychother.* 37 232–260. 10.1080/0075417X.2011.614738

[B28] Ornaghi V. Grazzani I. Zanetti M. A. (2010). Lessico psicologico e teoria della mente: uno studio con bambini di scuola primaria. *Età evolutiva* 97 54–71.

[B29] Pace C. S. Zavattini G. C. Tambelli R. (2015). Does family drawing assess attachment representations of late-adopted children? A preliminary report. *Child Adolesc. Ment. Health* 20 26–33. 10.1111/camh.12042 32680327

[B30] Russ S. (2004). *Play in Child Development and Psychotherapy: Toward Empirically Supported Practice.* Mahwah, NJ: Lawrence Erlbaum Associates.

[B31] Russ S. W. (1993). *Affect and Creativity: The Role of Affect and Play in the Creative Process.* Palo Alto, CA: Psychology Press.

[B32] Salcuni S. Capella C. Lis A. (2015). Introduction to the special issue on qualitative and quantitative research in child and adolescent psychotherapy. *Res. Psychother. Psychopathol. Process Outcome* 18 VIII–XI 10.7411/RP.2015.211

[B33] Shirk S. R. Karver M. (2003). Prediction of treatment outcome from relationship variables in child and adolescent therapy: a meta-analytic review. *J. Consult. Clin. Psychol.* 71 452–464. 10.1037/0022-006X.71.3.452 12795570

[B34] van Nijnatten C. van Doorn F. (2013). The role of play activities in facilitating child participation in psychotherapy. *Discourse Stud.* 15 761–775. 10.1177/1461445613490012

[B35] Weisz J. R. Hawley K. M. (2002). Developmental factors in the treatment of adolescents. *J. Consult. Clin. Psychol.* 70:21 10.1037/0022-006X.70.1.21 11860047

[B36] Winnicott D. W. (1942). “Why children play,” in *The Child and the Outside World: Studies in Developing Relationships*, ed. Hardenberg J. (London: Tavistock), 149–152.

[B37] Yanof J. A. (2013). Play technique in psychodynamic psychotherapy. *Child Adolesc. Psychiatr. Clin. N. Am* 22 261–282. 10.1016/j.chc.2012.12.002 23538013

